# Rare COVID-19 vaccine side effects got lost in the shuffle. Primary cutaneous lymphomas following COVID-19 vaccination: a systematic review

**DOI:** 10.3389/fmed.2024.1325478

**Published:** 2024-04-10

**Authors:** Berenika Olszewska, Anna Zaryczańska, Roman J. Nowicki, Małgorzata Sokołowska-Wojdyło

**Affiliations:** Department of Dermatology, Venereology and Allergology, Faculty of Medicine, Medical University of Gdańsk, Gdańsk, Poland

**Keywords:** SARS-CoV-2 mRNA vaccine, COVID-19, cutaneous lymphomas, side effects, SARS-CoV-2

## Abstract

**Introduction:**

COVID-19 vaccines are generally safe and effective; however, they are associated with various vaccine-induced cutaneous side effects. Several reported cases of primary cutaneous lymphomas (CLs) following the COVID-19 vaccination have raised concerns about a possible association. This systematic review aims to investigate and elucidate the potential link between CLs and SARS-CoV-2 vaccines.

**Methods:**

We performed a systematic literature search on PubMed, EBSCO and Scopus from January 01, 2019, to March 01, 2023, and analyzed studies based on determined eligibility criteria. The systematic review was performed based on the PRISMA protocol.

**Results:**

A total of 12 articles (encompassing 24 patients) were included in this analysis. The majority of CLs were indolent cutaneous T-cell lymphomas (CTCLs) (66,7%; 16/24), with Lymphomatoid papulosis (LyP) being the most common type (33,3%; 8/24). Most patients (79,2%; 19/24) developed lesions after receiving the COVID-19 mRNA-based vaccines, and predominantly after the first immunization dose (54,2%; 13/24). The presented CLs cases exhibited a tendency to exacerbate following subsequent COVID-19 vaccinations. Nevertheless, CLs were characterized by a favorable course, leading to remission in most cases.

**Conclusion:**

The available literature suggests an association between the occurrence and exacerbation of CLs with immune stimulation following COVID-19 vaccination. We hypothesize that post-vaccine CLs result from an interplay between cytokines and disrupted signaling pathways triggered by vaccine components, concurrently playing a pivotal role in the pathomechanism of CLs. However, establishing a definitive causal relationship between these events is currently challenging, primarily due to the relatively low rate of reported post-vaccine CLs. Nonetheless, these cases should not be disregarded, and patients with a history of lymphoproliferative disorders require post-COVID-19 vaccination monitoring to control the disease’s course.

Systematic review registrationwww.researchregistry.com, identifier [1723].

## Introduction

In March 2020, the World Health Organization (WHO) declared the novel coronavirus disease (COVID-19), caused by the severe acute respiratory syndrome coronavirus 2 (SARS-CoV-2) a global pandemic. According to the WHO COVID-19 dashboard, as of January 2024, over 770 million cases of COVID-19 have been confirmed, including more than 7 million deaths (1). The urgency of the pandemic required rapid development and introduction of vaccines, resulting in a relatively short follow-up period, which raised concerns about their safety. The mRNA-based vaccines (Pfizer/BioNTech, Moderna) were the first to be approved by US Food and Drug Administration (FDA) for preventing COVID-19 disease (2). Both mRNA vaccines demonstrated very high efficacy with mild to moderate adverse events (AEs) in the phase 3 randomized clinical trials (3, 4). The COVID-19 pandemic led to the development and approval of other vaccine types to control viral transmission. As of 8 April 2022, World Health Organization (WHO) has determined that the following authorized vaccines: inactivated-based vaccines (Sinovac, Covaxin, and Sinopharm), vector-based vaccines (AstraZeneca/Oxford, Johnson and Johnson, CanSino), mRNA-based vaccines (Pfizer/BioNTech, Moderna), and a subunit protein-based vaccine (Nuvaxovid and Covovax) against COVID-19 meet the required criteria for both safety and efficacy (5).

In response to the COVID-19 pandemic, mass vaccination programs have been implemented worldwide. To date, 67% of total population have been vaccinated with a complete primary series of a COVID-19 vaccine, and 32% with at least one booster dose (1). Consequently, there is a growing body of real-world evidence on AEs linked to the use of the COVID-19 vaccines. All available COVID-19 vaccines seem to be generally effective and safe; however, they are not devoid of side effects. According to data, the majority of side effects of mRNA COVID-19 vaccines are mild to moderate, including fever, fatigue, headache, muscle ache, and cutaneous manifestations at the injection site (3, 4, 6, 7). However, various rare cases of new-onset or flare of immune-mediated diseases, as well as hematologic malignancies and primary cutaneous lymphomas (CLs), have been reported (8–22).

The CLs represent a diverse group of non-Hodgkin lymphomas arising from T- or B-lymphocytes, primarily affecting the skin. They are classified as rare diseases, with estimated incidence rates ranging from 0.64 to 0.87 per 100,000 person-years, according to studies from the United States (23–25). Primary cutaneous T-cell lymphomas (CTCLs) constitute 75–80% of all CLs, while primary cutaneous B-cell lymphomas (CBCLs) constitute 20–25% (26, 27). The incidence rates vary geographically, with a slightly higher prevalence of CTCL in Asian and South American countries compared to Europe (28, 29). CLs are categorized into distinct subtypes that vary in terms of clinical presentation, behavior, histological features, and treatment. The clinical course of CLs is also highly variable, ranging from an indolent, slowly progressive course when the immune system controls tumor growth to aggressive forms with extracutaneous involvement and a poor prognosis (26). Mycosis fungoides (MF) and primary cutaneous CD30-positive lymphoproliferative disorders (CD30+ LPDs) account for nearly 80% of all CTCLs and are classified as indolent lymphomas (26, 27). Sézary syndrome (SS) represents the most common subtype among aggressive CTCLs, accounting for approximately 3% of all CTCLs (26, 27). MF and SS predominantly affect adults, with the peak incidence occurring in the sixth and seventh decades of life (26, 27). The male-to-female ratio also shows variability among different subtypes. MF typically presents as skin patches, plaques, and tumors, while SS is characterized by cutaneous involvement and a leukemic component. Other CTCLs are considered rare and collectively account for less than 10% of CTCLs cases (26).

The pathogenesis of CTCL is complex and not fully understood. The role of genetic, immunological, and environmental factors is being emphasized. Environmental mechanisms which may play a role in the evolution of CLs include long-term antigen stimulation by viral/microbial pathogens, drug triggers, geographic and occupational associations (30–33). The molecular and immunological processes lead to the clonal expansion of lymphocytes within the skin. Molecular alterations and immunological dysregulation, including impaired T-cell function, dysregulated cytokine signaling pathways, and altered cytokine profiles, play a pivotal role in driving malignant transformation, and disease progression (34, 35). Moreover, the interaction between malignant lymphocytes and the inflammatory microenvironment of the skin seems to be crucial in evading immune surveillance and sustaining the neoplastic process (35, 36).

In the context of CLs pathogenesis, the immunogenicity of COVID-19 vaccines appears to have the potential to influence the course of specific subset of the diseases, particularly lymphoproliferative disorders, including lymphomas such as CTCLs. However, there is limited evidence regarding the impact of vaccines on the cancer course in patients, particularly those with altered immunity due to lymphoproliferative malignancies. Therefore, the objective of the systematic literature review was to examine the association between COVID-19 vaccination and the occurrence or exacerbation of CLs.

## Materials and methods

### Search strategy and study selection

This study was conducted under the Guideline of Preferred Reporting Items Systematic Meta-Analyses Checklist (PRISMA) (37). The review protocol was registered at Research Registry (UIN: Review Registry 1723). The online search was conducted independently by two authors (B.O and A.Z.) on electronic websites, databases, and journals, including PubMed, Scopus, and EBSCO from January 01, 2019, to March 01, 2023. Discrepancies were solved by the third reviewer. Additionally, we manually screened references or citations of each article. The search was conducted using the combination of the following keywords and Medical Subject Heading (MeSH) terms: COVID vaccine, BNT162, ChAdOX1, AstraZeneca, mRNA-1273, cutaneous lymphoma, Lymphomatoid papulosis, Mycosis fungoides, Primary Cutaneous Anaplastic Large Cell Lymphoma, COVID-19, SARS-CoV-2.

**Figure 1:**
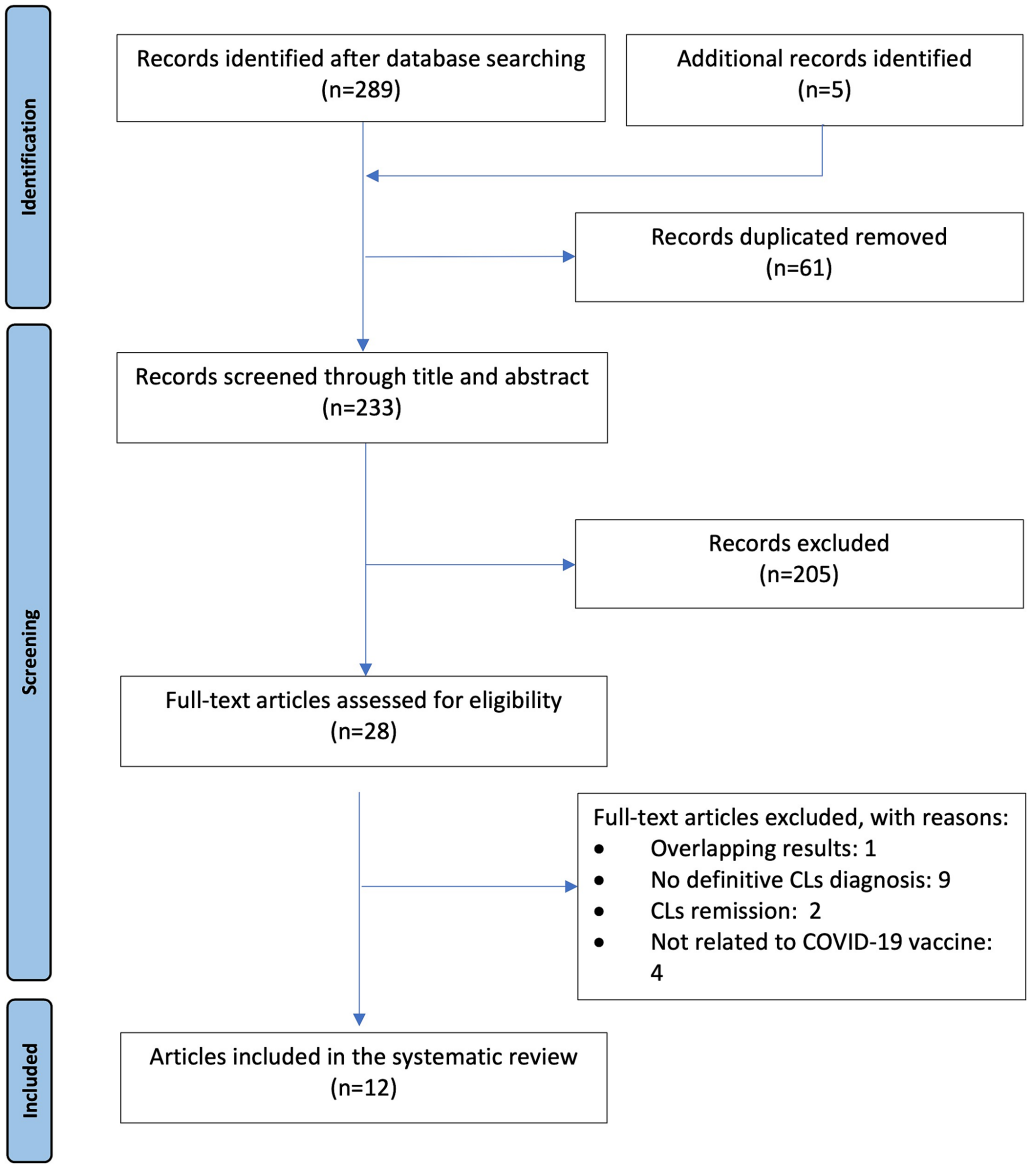
Flow diagram of the study according to PRISMA (37).

Inclusion criteria were studies describing patients with a definitive diagnosis of CLs who experienced onset, relapse, or exacerbation after immunization with a COVID-19 vaccine with WHO Emergency Use Listing. Case reports, letters to the editor, conference abstracts and case series were included. Articles involving children (<18 years), reviews, duplicate studies, personal experience summaries, lymphomas other than primary cutaneous, resolution of CLs, doubtful diagnosis of CLs, studies not meeting the inclusion criteria of this study or in a language other than English were excluded. Initial screening involved the evaluation of titles and abstracts, followed by a full-text assessment for eligibility. Additionally, references cited in relevant papers were also followed up for additional studies. The PRISMA flow diagram of the search method used in this systematic review is presented in Figure 1.

### Data extraction and data synthesis

Two researchers (B.O. and A.Z.) extracted the following information from full-text articles: first author (reference); age; sex; SARS-CoV-2 vaccine type and doses administered; the time between administration and lesions onset; definitive diagnosis before and after vaccination; management; outcomes. The selected articles were double-checked by other researchers. A narrative synthesis was performed, and data focusing on population, intervention, comparison and outcome were synthesized through descriptive statistical analyses using Microsoft Excel software.

### Quality assessment

The quality of case reports and case series included in the systematic review was assessed using the Joanna Briggs Institute Critical Appraisal Checklist for Case Reports and Case Series (38). The overall quality of the included studies was assessed: A “low risk” of bias score was defined when responses of “yes” to all of the applicable questions was provided. When at least one answer to applicable questions was found “unclear,” a scoring of “moderate risk” was defined. The response of “no” to at least one of the questions rendered it to be of “high risk” of bias.

## Results

We identified potential 294 records, 61 duplicates were excluded, 204 were excluded after the title and abstract screening and 17 were excluded after the full-text screening. Finally, 12 articles met the eligibility criteria for inclusion in the systematic review. The majority of publications were case reports (*n* = 5) and letters to the editor (*n* = 5), followed by research letter (*n* = 1) and conference abstract (*n* = 1). The cohort comprised 24 patients, including 15 males and 9 females, with a median age of 60.5 years (range, 20–80 years). All 24 patients were diagnosed with CLs after COVID-19 vaccination, all of which were CTCLs. The details of each case are presented in the Table 1.

**Table 1:** Characteristics of the studies reporting primary cutaneous lymphomas following COVID-19 vaccination.

No./reference	Age/gender	Time from vaccination to onset of lesions	Type and dose of vaccine	Type of CLs before vaccination	HP examination after vaccination	CD30	Stage before vaccination	Course of CLs after vaccinations	Treatment and outcome
1 (11)	70/M	2 days after 1st dose	mRNA vaccine-BNT162b2	pcALCL	pcALCL	+	CR	Relapse	SR
2 (12)	60/M	4 weeks after 1st dose	Viral vector- AZD122	Folliculotropic MF-early stage	CD30+ LCT-MF tumor stage	+	R	Exacerbation; Progression after 2 dose	?
3 (12)	73/F	10 days after 1st dose	Viral vector- AZD122	MF – early stage and LyP type-A	LyP type-A	+	R	Relapse	?
4 (13)	60/M	7 days after 1 st dose	Viral vector - AZD122	−	LyP type- D	+	−	New-onset	SR
5 (13)	66/F	10 days after 1 st dose	mRNA vaccine-BNT162b2	−	Ly type-D	+	−	New-onset; Exacerbation after 2 dose	NB-UVB – CR, recurrence, MTX- current treatment
6 (14)	56/F	2 days after 1st dose	mRNA vaccine-BNT162b2 2 dose; mRNA1273	−	CD8+ MF	?	−	New-onset; Exacerbation after 2 dose	TCS – CR
7 (15)	28/F	Few days after 1st dose	Viral vector– Ad26.COV2.S	−	SPTCL	−	−	New-onset	CsA, SCS – CR (with atrophy)
8 (16)	79/M	3 days after vaccine booster	mRNA vaccine booster- mRN-1273	−	PCGD-TCL	−	−	New-onset	Surgical excision, RT- CR
9 (17)	53/M	3 days after 1st dose	mRNA vaccine- BNT162b2	−	pcENKTL	+	−	New-onset; Exacerbation after 2 dose	CHT, RT-?
10 (18)	76/M	10 days after vaccine booster	mRNA vaccine booster- mRN-1273	-	PC-ALCL	+	−	New-onset	SR
11 (19)	62/F	Several days after 2nd dose	mRNA vaccine- BNT162b2	−	CD8+ pcPTL-NOS	+/−	−	New-onset; progression after SARS-CoV-2 infection	TCS, SCS, MTX, BV- Lack of response
12 (20)	50/M	4 days after 1st dose	mRNA vaccine- BNT162b2	−	LyP type-A	−	−	New-onset	MTX- CR
13 (20)	20/F	42 days after 1 dose	mRNA vaccine- BNT162b2	−	LyP type-A	+	−	New-onset	MTX- CR
14 (21)	79/M	30 days after 1st dose	Inactivated SARSCoV2 viral vaccine- CoronaVac (Sinovac)	−	pcPTL-NOS	−	−	New-onset; exacerbation after 2 nd (inactivated virus vaccine) and 3 rd dose (recombinant adenovirus mechanism)	?
15 (22)	67/M	15 days after 2nd dose	mRNA vaccine-BNT162b2	LyP type-A diagnosed in 2019, relapsed in 2020	LyP type-A	? (+)	−	Relapse	MTX- CR
16 (22)	49/M	15 days after 2nd dose	mRNA vaccine-BNT162b2	CD4+ PCSM-LPD	CD4+ PCSM-LPD	?	CR	Relapse	SR
17 (22)	58/M	2 days after 2nd dose	mRNA vaccine-BNT162b	SS	SS	?	CR	Relapse	TCS, SCS, MOGA – CR
18 (22)	61/M	14 days after 3rd dose	mRNA vaccine-BNT162b	MF-early-stage	Erythrodermic MF	?	SD	Progression	OCS, PUVA - CR
19 (22)	61/M	15 days after 3rd dose	mRNA vaccine-BNT162b	SS	SS	?	Well-managed	Relapse	ECP- PR
20 (22)	80/F	15 days after 3rd dose	mRNA vaccine-BNT162b	−	SS	?	−	New-onset	TCS, SCS- CR
21 (22)	60/M	30 days after 2nd dose	mRNA vaccine-BNT162b	−	LyP type- A	? (+)	−	New-onset	SCS, Trimeton- CR
22 (22)	52/F	3 days after 1st dose	mRNA vaccine-BNT162b	−	CD4+ PCSM-LPD	?	−	New- onset	RT- CR
23 (22)	61/F	10 days after 1st dose	mRNA vaccine-BNT162b	−	LyP type- A	? (+)	−	New- onset	SR
24 (22)	45/M	20 days after 3rd dose	mRNA vaccine- BNT162b	−	CD4+ PCSM-LPD	?	−	New- onset	Surgical excision – CR

Ad26.COV2.S, Janssen vaccine; AZD122, AstraZeneca vaccine; BNT162b2, BioNTech, Pfizer COVID-19 mRNA vaccine; BV, brentuximab vedotin; CD4+ PCSM-LPD, CD4+ primary cutaneous small/medium T-cell lymphoproliferative disorder; CHT, chemotherapy; CR, complete remission; CsA, cyclosporin; ECP, extracorporeal photopheresis; LyP, lymphomatoid papulosis; MF, mycosis fungoides; MOGA, mogamulizumab; mRNA1273, Moderna COVID-19 vaccine; MTX, methotrexate; pcALCL, primary cutaneous anaplastic large cell lymphoma; PCGD-TCL, primary cutaneous γ/δ T-cell lymphoma; pcENKTL, primary cutaneous extranodal NK/T-cell lymphoma; pcPTL-NOS, primary cutaneous peripheral T-cell lymphoma, not otherwise specified; R, remission; RT, radiotherapy; SCS, systemic corticosteroids; SD, stable disease; SPTCL, Subcutaneous panniculitis-like T-cell lymphoma; SR, spontaneous remission; SS, Sézary syndrome; TCS, topical corticosteroids;?: data not provided.

**Figure 2:**
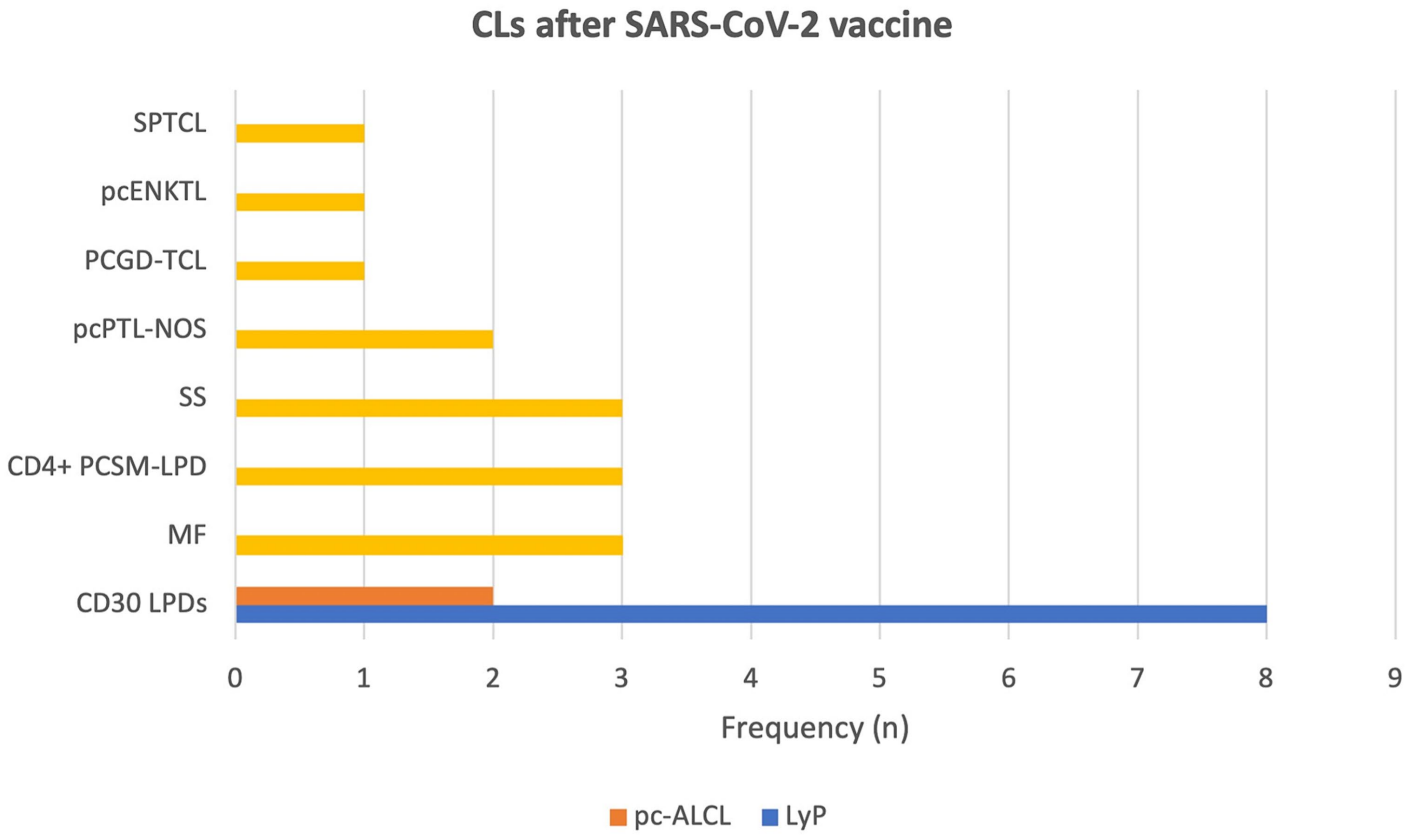
Graphical representation of frequencies in reported types of primary cutaneous lymphomas after SARS-CoV-2 vaccines. (CD4+ PCSM-LPD, CD4+ primary cutaneous small/medium T-cell lymphoproliferative disorder; LyP, lymphomatoid papulosis; MF, mycosis fungoides; pcALCL, primary cutaneous anaplastic large cell lymphoma; PCGD-TCL, primary cutaneous γ/δ T-cell lymphoma; pcENKTL, primary cutaneous extranodal NK/T-cell lymphoma; pcPTL-NOS, primary cutaneous peripheral T-cell lymphoma, not otherwise specified; SPTCL, subcutaneous panniculitis-like T-cell lymphoma; SR, spontaneous remission; SS, Sézary syndrome).

The majority of reported CLs were indolent CTCLs (66,7%; 16/24), followed by aggressive CTCLs (33,3%; 8/24). CD30+ LPDs were the most frequently reported subgroup of CTCLs (41,7%; 10/24) with LyP being the most common type (33,3%; 8/24) (12, 13, 20, 22) followed by primary cutaneous anaplastic large cell lymphoma (pc-ALCL) (8,3%; 2/24) (11, 18). Other reported CLs were 2 cases of MF (14, 22) including CD8+ MF, 3 cases of primary cutaneous CD4+ small/medium T-cell lymphoproliferative disorder (CD4+ PCSM-LPD) (22) and single case of subcutaneous panniculitis-like T-cell lymphoma (SPTCL) (15). Reported cases of aggressive CTCLs included 3 cases of SS (22), 2 primary cutaneous peripheral T-cell lymphomas, not otherwise specified (pcPTL-NOS) (19, 21) followed by single case of primary cutaneous γ/δ T-cell lymphoma (PCGD-TCL) (16), mycosis fungoides with large cell transformation (MF-LCT) (12) and primary cutaneous (extranodal) NK/T-cell lymphoma (pcENKTL) (17). The summarized data are presented in Figure 2.

**Figure 3:**
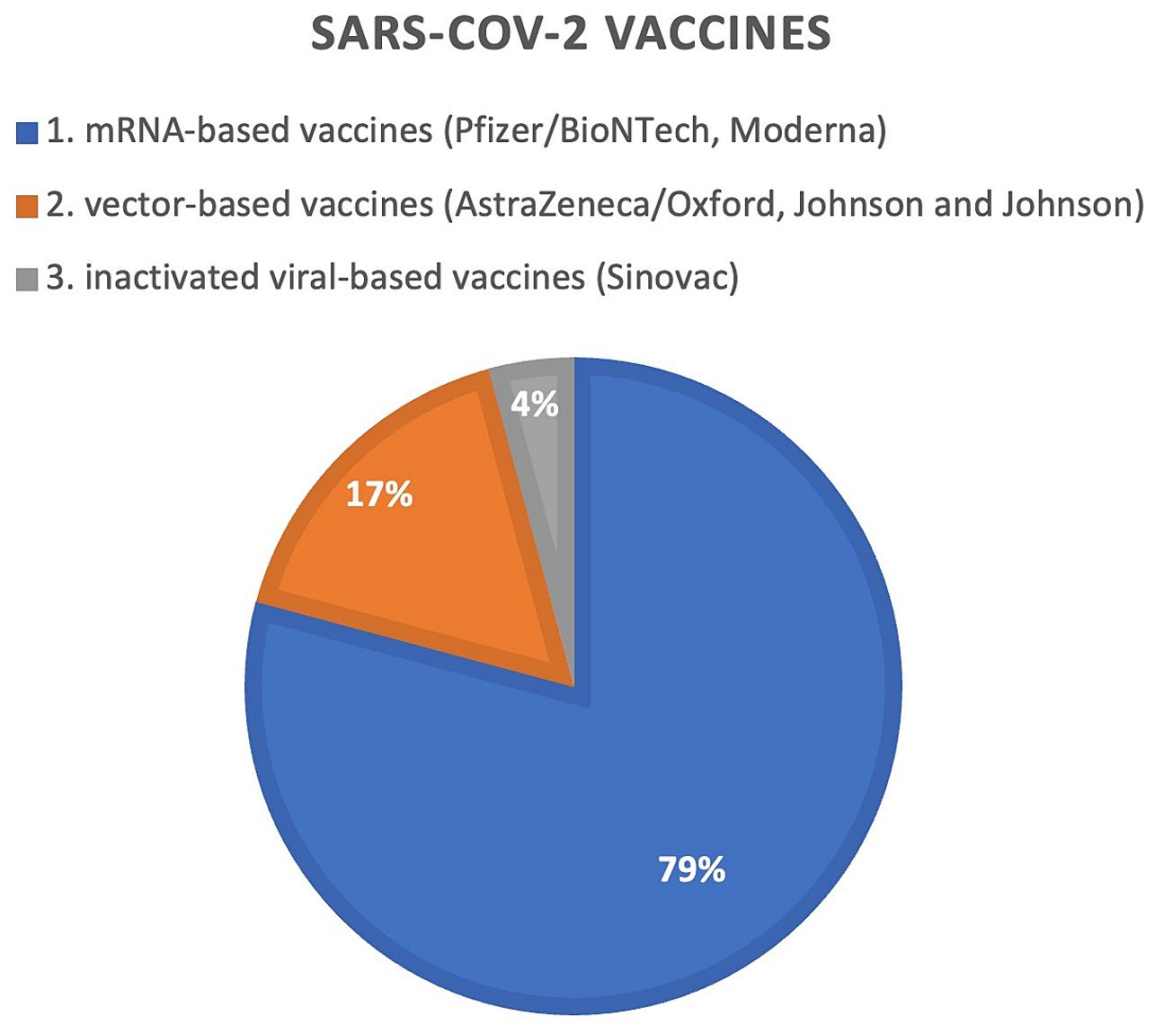
Graphical representation of frequencies in reported SARS-CoV2 vaccines inducing primary cutaneous lymphomas.

The available data are limited, however, histologic examination revealed a predominant feature of T-cell phenotype, 12 out of 16 (75%) reported cases presented expression of CD30+ antigen (11–13, 17–20, 22). In 8 cases, data regarding CD30 expression in histopathology were missing (14, 22). The vast majority of patients (79,2%; 19/24) developed lesions after receiving COVID-19 mRNA-based vaccines, followed by vector-based vaccines (16,7%; 4/24) and inactivated SARSCoV2 viral vaccine (4,1%; 1/24). We have summarized the data in Figure 3. More than half (66,6%; 16/24) of the patients received the Pfizer/BioNTech vaccine (11, 13, 14, 17, 19, 20, 22), while the rest got AstraZeneca/Oxford (12,5%; 3/24) (12, 13), Moderna (12,5%; 3/24) (14, 16, 18), Johnson and Johnson (4,2%; 1/24) (15) and Sinovac (4,2%; 1/24) (21). The majority of cases (66,7%; 16/24) (13–22) were new-onsets of CLs, while the rest of the cases were exacerbation/progression (8,3%; 2/24) (12, 22) and recurrence of CLs (25%; 6/24) (11, 12, 22). Most of the cases (54,2%; 13/24) were recorded after the first immunization dose (11–15, 17, 20–22), followed by 5 cases that developed after the second immunization dose (20,8%; 5/24) (19, 22) and 6 after the third (booster) COVID-19 vaccine dose (25%; 6/24) (16, 18, 22). Five studies provided data regarding the deterioration of lesions following second and subsequent COVID-19 vaccinations (12–14, 17, 21). The median time from vaccination to symptom onset was 10 days (ranging from 2 to 42 days).

Treatment of CLs following COVID-19 vaccination comprised systemic, topical treatment, and combination. Five out of 21 (24%) recorded cases experienced spontaneous remission (11, 13, 18, 22). Overall, 16 patients needed systemic treatment, including methotrexate (MTX), cyclosporin (CsA), brentuximab vedotin (BV), mogamulizumab (MOGA), systemic corticosteroids (SCS), chemotherapy (CHT) and extracorporeal photopheresis (ECP). Local treatment methods included corticosteroids (TCS), radiotherapy (RT), and surgical excision of lesions. The majority of CLs cases achieved complete remission (CR) (14–16, 20, 22) or partial remission (PR) (22) following standard treatment. One case achieved remission with subsequent relapse of disease (13), and one did not respond to therapy (19). In three cases, data concerning treatment outcome were incomplete (12, 17, 21).

### Quality assessment of included studies

Most of the studies were assessed as low (11, 14–16, 18, 20, 22) or moderate risk of bias (12, 17, 21), mainly due to incomplete treatment outcome data (Supplementary Tables S1, S2).

## Discussion

Since the global introduction of vaccination programs, our understanding of COVID-19 vaccine-related cutaneous reactions is continually expanding. Numerous diverse cutaneous reactions following COVID-19 vaccination have been reported, whereby some of them appear to have an immunological or autoimmunological background. According to the available data, the predominant cutaneous side effect associated with SARS-CoV-2 vaccination is a mild and self-limited local injection-site reaction, followed by unspecified cutaneous eruptions, urticaria, angioedema, herpes zoster, pityriasis rosea-like eruptions, pernio, vasculitis, morbilliform eruption and facial dermal filler reactions (39–44). Additionally, rare cutaneous AEs such as the new onset or exacerbation of autoimmune blistering disease, psoriasis, atopic dermatitis, eczema, lichen planus, cutaneous lupus erythematosus, as well as the new onset or recurrence of lymphoproliferative disorders, have been reported (11–22, 39–44).

Upon completing the analysis of the 24 CLs after COVID-19 vaccination, several observations can be drawn regarding a possible association between these events. In this systematic review of case reports and case series, we found that CD30 LPDs, namely LyP and PC-ALCL, were the most frequently reported CLs after immunization with a SARS-CoV-2 vaccine. However, marked positive expression of CD30 antigen was also noted in MF, pcENKTL, and PCGD-TCL. Most cases occurred after the administration of COVID-19 mRNA-based vaccines, with the majority of CLs being triggered by the first immunization dose and were newly diagnosed. At the same time, the presented cases of CLs showed a tendency to exacerbate following the second and subsequent administrations of COVID-19 vaccine. The disease courses were rather favorable resulting in remission following standard treatment in the majority of cases, including aggressive CTCLs. Moreover, approximately one-quarter of the described patients experienced spontaneous resolution of lesions.

The observed predominance of CD30+ positive cutaneous lymphomas induced by COVID-19 vaccinations raises the question of whether the COVID-19 vaccine might induce the proliferation of CD30+ T-cells in patients with active disease. The CD30 antigen is expressed on a small subset of activated T and B lymphocytes in hematopoietic malignancies, including Hodgkin lymphoma and CTCL (45). Antigenic stimulation by mitogens and viruses has been demonstrated to drive CD30 expression on lymphocytes (46). Moreover, a highly potent adaptive immune response after repeated immunizations with COVID-19 vaccines is suggested to trigger immune exhaustion, leading to the depletion of both CD4+ and CD8+ T-cells, which exhibit altered or diminished effector functions against both tumor antigens and pathogens (47). It is particularly interesting since exhaustion of activated T lymphocytes is a feature of both CD4+ and CD8+ T cells isolated from advanced CTCL skin lesions (48). There have been suggestions that the recurrence of lymphomas is linked to mRNA COVID-19 vaccines, possibly due to immune system overstimulation, leading to viral-associated CD30 expression and subsequent exhaustion of T-cells (11, 12). We hypothesize that overproduction and exhaustion of CD4+/CD8+ T cells expressing CD30 may lead to evasion of immune surveillance, thereby contributing to the exacerbation or development of CLs.

Another possible explanation for newly diagnosed and relapsed CLs after COVID-19 vaccinations is that the vaccines might stimulate signaling pathways that drive the pathogenesis. CLs were reported after immunization with both mRNA and vector-based vaccines. However, most of reported cases were induced by lipid nanoparticles (LNPs) formulated messenger RNA-based (LNP-mRNA) COVID-19 vaccines. We suspect that it might be partially related to the LNPs carrier. According to the literature, all components of mRNA COVID-19 vaccines, including LNPs, mRNA, and the produced antigen- S protein, may trigger proinflammatory action (49). However, there is robust evidence of the highly inflammatory properties of LNPs, resulting in stronger adjuvant activity compared to other adjuvants (50–52). Mouse models have shown that LNPs induce an inflammatory milieu characterized by neutrophil infiltration, activation of various inflammatory pathways, and production of inflammatory cytokines and chemokines that might be responsible for reported side effects (50). LNPs were also demonstrated to exacerbate already existing inflammation in mouse models (51). In addition, LNPs and mRNA were shown to activate various Toll-like receptors (TLRs) that trigger signaling pathways involved in immune defense against pathogens (53–55). Interestingly, mRNA COVID-19 vaccine was demonstrated to activate immune cells via TLR3, leading to the secretion of IL-6 and subsequent STAT3 phosphorylation (56). Whereby, IL-6 is a common activator of both NF-KB and STAT3 signaling pathways (57) and has been found to be overexpressed in CTCL (58).

Apart from LNPs and mRNA, the SARS-CoV-2 S1 spike protein induces an excessive inflammatory response. Interestingly, Cheng et al. (59) demonstrated that S1 protein has a unique superantigen-like motif which is highly similar to the bacterial superantigen staphylococcal enterotoxin (SE). Therefore, the SARS-CoV-2 S protein is suspected to have potent superantigen properties and to act similarly to bacterial superantigens, thus influencing T cell repertoire (59). This might be significant in terms of lymphomas, as SE are believed to induce disease activity in CLs (60). Moreover, several studies have reported that the SARS-CoV-2 S1 spike protein acts by inducing the production of inflammatory cytokines and chemokines (TNF-alfa, IL-6, IFN gamma) and activating various pathways (ERK1/2 MAPK, NF-kB) (61–63). Therefore, AEs are suspected to be linked to vaccine synthesized SARS-CoV-2 spike proteins, as they may affect host cells in a similar way to COVID-19 infection (64). Taken together, immunization with the mRNA COVID-19 vaccine may trigger overstimulation of the IL-6/STAT3/NFkB loop. This finding is crucial when considering its impact on the CTCL course. Our findings suggest that, although COVID-19 vaccination may elicit CLs, it is not associated with an aggressive clinical course or resistance to treatment. The majority of reported CLs cases showed a very good response to standard treatment, leading to the remission of lesions, even in cases of aggressive CLs.

Notably, new onsets and relapses of CLs have been described following COVID-19 vaccination, but the exact pathogenic mechanism is not fully understood. The predominance of newly diagnosed CLs after vaccination raises the question of whether SARS-CoV-2 vaccine may elicit oncogenesis. There is too little data available to assume, with certainty, that COVID-19 vaccines may contribute to CLs occurrence. However, we suspect that Covid-19 vaccines have the potential to unmask the sub-clinical lymphoproliferative disorders rather than initiate tumorgenesis. It is probable that the newly diagnosed cases had smoldering lymphoproliferation that was controlled by immune surveillance, while vaccination created favorable conditions for the outbreak of the disease. It appears that SARS-CoV-2 vaccines may drive the modification of cytokine profiles in the skin milieu, exacerbate pre-existing inflammation, and activate diverse signaling pathways, potentially leading to either exacerbation or even resolution of the disease.

Nevertheless, it is crucial to emphasize that COVID-19 vaccines are generally safe and highly effective in preventing severe outcomes from COVID-19 infection. Moreover, there is compelling evidence indicating their benefits for patients with solid cancers and those on immunosuppressive treatment (65–67). Notably, two exceptional cases have been reported, demonstrating spontaneous regression of primary cutaneous follicle center cell lymphoma and resolution of organ involvement in PC-ALCL after COVID-19 vaccination (68, 69). These cases were not included in the systematic review as they met exclusion criteria. Such surprising observations suggest potent modulatory properties of COVID-19 vaccination, potentially enhancing the anti-tumor response in predisposed individuals (68).

### Limitations

The limitations of this report include the restricted number of studies retrieved from the literature, despite a thorough literature search. This limitation arises from the fact that CLs are rare diseases. However, the findings of this study provide potentially valuable information about rare vaccine-related cutaneous reactions. Moreover, the available data on SARS-CoV-2 vaccine-related CLs primarily originate from case reports and case series, limiting the ability to assess incidence rates of these side effects. Additionally, the collected data were diverse and sometimes incomplete thus precluding meta-analysis, which might constitute the biggest limitation of this study. However, it should be stressed that the presented systematic review is the first to analyze and summarize available literature data on CLs occurring after the administration of the SARS-CoV-2 vaccine. In addition, due to potential underreporting of side effects such as CLs following immunization with COVID-19 vaccine, clinical trials are still needed to investigate the potential correlation between vaccines and lymphoproliferative disorders.

## Conclusion

In this systematic review, we analyzed the cases of CLs occurrence or exacerbation following COVID-19 vaccination. Given the scarce data, establishing a definitive causal relationship between COVID-19 immunization and an increased risk of lymphoma development or exacerbation is challenging. Nonetheless, the striking similarities observed in the reported post-vaccine CLs cases should not be underestimated. The literature review highlights the potent stimulation of immune cells that may result in a flare-up or onset of post-vaccine CLs, particularly in susceptible populations. We believe that the components of COVID-19 vaccines may modulate the microenvironment of CLs leading to the exacerbation or outbreak of sub-clinical cutaneous lymphoproliferation. Further studies are needed to verify and understand the potential relationship between CLs and vaccination. Until then, patients with a history of lymphoproliferative disorders should always be carefully followed-up to monitor the disease course after COVID-19 vaccination.

## Data availability statement

The original contributions presented in the study are included in the article/Supplementary material, further inquiries can be directed to the corresponding author.

## Author contributions

BO: Conceptualization, Methodology, Data curation, Investigation, Writing – original draft. AZ: Data curation, Conceptualization, Investigation, Writing – original draft. RN: Writing – review & editing. MS-W: Writing – review & editing.
