# Supplementary material for: Rare COVID-19 vaccine side effects got lost in the shuffle. Primary cutaneous lymphomas following COVID-19 vaccination: a systematic review
Source: Front Med (Lausanne). 2024 Apr 10;11:1325478. doi: 10.3389/fmed.2024.1325478 (PMC11041019; doi:10.3389/fmed.2024.1325478)
Supplement: Supplementary file 1 [file Table_1.DOCX]

Supplementary Table 1. JBI Critical Appraisal Checklist for Case Reports

| JBI checklist questions | [11]  Brumfiel  Et al 2021 | [12]  Panou et al 2022 | [13]  Koumaki et al 2022 | [14]  Li et al 2022 | [15]  Kreher  et al  2022 | [16]  Hobayan et al 2023 | [17]  Zamfir et al 2022 | [18]  Revenga-Porcel et al 2023 | [19]  Bresler  et al 2023 | [20]  Hooper et al  2022 | [21]  Montoya  et al 2022 |
| --- | --- | --- | --- | --- | --- | --- | --- | --- | --- | --- | --- |
| Were patient’s demographic characteristics clearly described?  Was the patient’s history clearly described and presented as a timeline?  Was the current clinical condition of the patient on presentation clearly described?  Were diagnostic tests or assessment methods and the results clearly described?  Was the intervention(s) or treatment procedure(s) clearly described?  Was the post-intervention clinical condition clearly described?  Were adverse events (harms) or unanticipated events identified and described?  Does the case report provide takeaway lessons? | YES  YES  YES  YES  YES  YES  YES  YES | YES  YES  YES  YES  YES  UNC  YES  YES | YES  YES  YES  YES  YES  YES  YES  YES | YES  YES  YES  YES  YES  YES  YES  YES | YES  YES  YES  YES  YES  YES  YES  YES | YES  YES  YES  YES  YES  YES  YES  YES | YES  YES  YES  YES  YES  UNC  YES  YES | YES  YES  YES  YES  YES  YES  YES  YES | YES  YES  YES  YES  YES  YES  YES  YES | YES  YES  YES  YES  YES  YES  YES  YES | YES  YES  YES  YES  YES  UNC  YES  YES |

Yes; No; UNC : Unclear; NA: Not applicable

Supplementary Table 2. JBI Critical Appraisal Checklist for Case Series

| JBI checklist questions | [22] Avallone et al 2023 |
| --- | --- |
| Were there clear criteria for inclusion in the case series?  Was the condition measured in a standard, reliable way for all participants included in the case series?  Were valid methods used for identification of the condition for all participants included in the case series?  Did the case series have consecutive inclusion of participants?  Did the case series have complete inclusion of participants?  Was there clear reporting of the demographics of the participants in the study?  Was there clear reporting of clinical information of the participants?  Were the outcomes or follow up results of cases clearly reported?  Was there clear reporting of the presenting site(s)/clinic(s) demographic information?  Was statistical analysis appropriate? | YES  YES  YES  YES  YES  YES  YES  YES  NA |

Yes; No; UNC : Unclear; NA: Not applicable
